# PrEP and choice counselling – Insights into implementation

**DOI:** 10.4102/jphia.v17i1.1458

**Published:** 2026-02-17

**Authors:** Melanie Pleaner, Faith Musvipwa, Siphokazi Dada, Fatima A. Cholo, Catherine E. Martin, Alison Kutywayo, Vusile Butler, Saiqa Mullick

**Affiliations:** 1 Wits Reproductive Health and HIV Institute, Faculty of Health Sciences, University of the Witwatersrand, Johannesburg, South Africa

**Keywords:** pre-exposure prophylaxis, PrEP choice, choice counselling, HIV prevention, training, transfer of learning

## Abstract

Adolescent girls and young women are disproportionately affected by human immunodeficiency virus (HIV). The introduction of oral pre-exposure prophylaxis (PrEP) was a game-changer in HIV prevention. In 2023, the dapivirine vaginal ring (DVR) was introduced in select implementation research sites in South Africa, introducing a new dimension into HIV prevention service delivery – people were now able to choose their PrEP method and switch between methods. As new PrEP products are introduced, choice has taken centre stage in HIV prevention service delivery. In these field notes, we describe an iterative, participative process to gain insight into choice counselling training and implementation. Healthcare providers were trained on both DVR and PrEP choice counselling. Guided by the Transfer of Learning Matrix, we embarked on a process with the aim of assessing how choice counselling was being implemented at site level to identify gaps and inform future training and mentoring in relation to effective choice counselling. Healthcare providers involved in delivering DVR in the three implementing clusters were purposefully invited to participate in interviews. A total of 42 interviews were conducted with both clinical and non-clinical providers between December 2023 and January 2024. All interviews were audio-recorded, transcribed verbatim and analysed thematically.

**Contribution:**

Findings include the importance of healthcare providers communicating in a balanced manner and not letting personal bias interfere, and that one-off trainings and job aids for choice counselling are not enough and need to be supplemented by active engagement with healthcare providers on the ground to identify gaps and inform ongoing in-service training and mentoring.

## Introduction

Human immunodeficiency virus (HIV) prevalence in South Africa remains high, with 7.7 million people living with HIV in 2023.^1^ Women, particularly adolescent girls and young women (AGYW) aged 15–24 years, are disproportionately affected – HIV prevalence in AGYW is three times higher than their male counterparts.^2^

A major turning point in HIV prevention in South Africa was the introduction of oral pre-exposure prophylaxis (PrEP) in 2016. Although oral PrEP has been a game-changer, challenges in real life implementation are evident including factors such as remembering to take the pill daily, the pill size, fear of others finding out, stigma and poor continuation.^3^ In addition, healthcare providers have ambivalent perceptions including concerns about PrEP resulting in disinhibition and abandoning condom use.^4^

The introduction of the dapivirine vaginal ring (DVR) in 2023 ushered in a new and exciting era in HIV prevention options in South Africa. Clients were now able to choose their PrEP method with both oral PrEP and the DVR available at select implementation study sites. The process of informed decision-making and choice in the context of PrEP is an important but new and complex process – both for healthcare providers and for clients, and even more so as we pave the way for the introduction of long-acting injectable methods such as cabotegravir (CAB-LA) and lenacapavir (LEN).^5^ Choice introduces a new dimension to PrEP service delivery.^6^ Evidence drawn from contraception tells us that choice enhances product utilisation and continuation.^7^ From the PrEP field, there is emerging evidence that choice of PrEP methods can increase prevention coverage and reduce the incidence of HIV.^8^

In this report, we describe one component of an *iterative participatory approach* used to support healthcare providers with the introduction of new PrEP methods and choice counselling. Drawing from the Transfer of Learning framework,^9,10^ we embarked on a process with the aim of assessing the implementation of choice counselling after training, in order to identify gaps and inform future training and mentoring.

## Methods

### Programme description

Project PrEP (funded by Unitaid), initiated in 2018, is a South African based implementation science study, aiming to inform the introduction of PrEP, coupled with integrated sexual and reproductive health (SRH) services, with a focus on AGYW.^11^ As an implementation science project, the focus has been on research and evaluation of strategies for demand generation, training and mentorship, and different platforms for service delivery to improve access and support effective PrEP use.^11^ After DVR and CAB-LA were introduced in August 2023 and April 2024 respectively, the focus expanded to include PrEP choice and decentralised care into community-based service delivery points. Project PrEP has a national office (headquarters) which provides leadership, technical expertise and coordination for the project’s implementation, including research, training, communication and mentoring. The eight project sites are located in three provinces, each comprising two fixed public healthcare clinics and a mobile clinic which provides community-based services (in Gqeberha, Eastern Cape; Mthatha, Eastern Cape; eThekwini, KwaZulu-Natal; and Tshwane, Gauteng).

In this report, we describe the work done in three of the four areas (i.e. Gqeberha, Mthatha, and Tshwane), which have been offering DVR in addition to oral PrEP, and planning for the introduction of CAB-LA.

### Our approach: Iterative, participatory methodology and transfer of learning matrix

In preparation for the pending arrival of the DVR and CAB-LA in South Africa, we wanted to gain insight into healthcare providers’ perceptions about these new biomedical prevention methods. We developed a human centred design (HCD) process^12,13^ and a workshop was conducted on 23 July 2023 – 24 July 2023 with nine healthcare providers. We used the insights from this workshop to guide the development of training materials and job aids. Before the delivery of the DVR to project sites, training was conducted with healthcare providers. Topics included clinical management of the DVR and choice counselling techniques. The introduction of DVR as a PrEP option commenced at project sites in August 2023.

**FIGURE 1:**
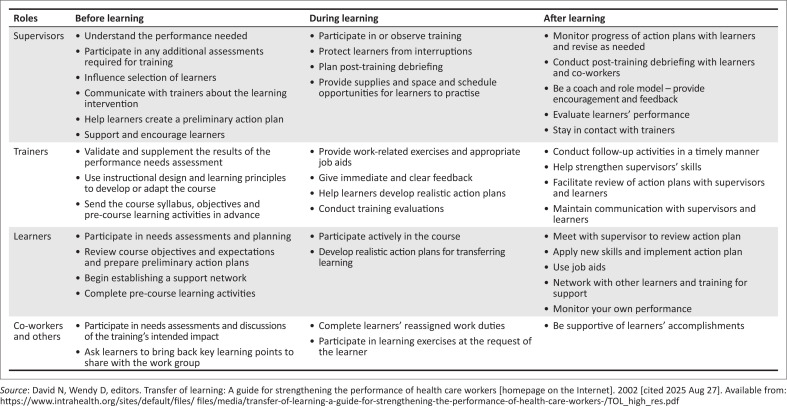
Transfer of learning matrix.

Three months into the introduction of DVR, we embarked on a process at respective project sites to gain insight and assess how informed decision-making and choice counselling were being implemented on the ground, and importantly, to inform future training, mentoring and support needs. The Transfer of Learning Matrix^10^ (Figure 1) provided a useful framework to guide our post-training assessment. Based on research from trainers, supervisors and learners in the health field, the Transfer of Learning Matrix outlines important actions that can be taken before, during, and after training to strengthen support for the transfer of knowledge and skills and to improve work performance.^10^

### Purpose

The purpose of these site assessment visits was to understand how the knowledge and skills imparted during the training were being applied in relation to choice counselling, especially because training took place prior to the arrival of the DVR and that choice counselling was a new dimension to PrEP provision. Through interactive engagement with healthcare providers, we were interested in learning about their experiences with choice counselling; their interpretation of choice; respective team members’ roles in providing information and counselling about the PrEP methods, including the newly introduced DVR, and in assessing what further training and support is required to enhance PrEP choice, thereby providing guidance to both supervisors, project mentors and trainers.

### Research methodology

The site engagements took place three months after the DVR was introduced between December 2023 and January 2024.

Individual and group interviews were conducted with 43 clinical (professional nurses) and non-clinical (lay counsellors, client navigators and fieldworkers) healthcare providers (Table 1). Participants were purposively sampled to include those providing new PrEP methods and choice counselling. Research staff invited potential participants via phone and email, explained the study to them, and, if they expressed interest, obtained their informed consent to participate.

**TABLE 1:** Description of participants (*N* = 43).

Characteristics	*n*
**Age group (years)**	
20–29	10
30–39	21
40–49	10
≥ 50	2
**Sex**	
Female	36
Male	7
**Cluster**	
Mthatha	14
Tshwane	15
Gqerbeha	14
**Current position**	
Professional nurses	11
Lay counsellors	7
Fieldworkers	12
Client navigators	12
Training manager	1
**Responsibilities related to PrEP provision (multiple responses)**	
Prescribing	12
Counselling	19
Community outreach	12
Education	43

A semi-structured interview guide was used to guide the discussions. In addition, participants were given a set of nine cards and requested to arrange, in order of importance, the factors they felt to be most influential of clients’ choice of PrEP methods. Interviews were audio-recorded supplemented by note taking, transcribed verbatim and analysed thematically to inform the summary of key learnings and recommendations. Data were managed and coded inductively using NVivo 14 software. Two researchers independently coded two transcripts, compared results to ensure consistency and collaboratively developed a coding framework. This framework was refined through discussion and then systematically applied to all transcripts for thematic analysis.

Feedback sessions were convened to share findings with key managers and staff responsible for mentoring and training.

### Ethical considerations

This workshop formed part of a series of formative work approved by the Human Research Ethics Committee of the University of the Witwatersrand (No. M210849) and the World Health Organization (WHO) Ethics Review Committee. Participation was voluntary, and all participants provided written informed consent.

## Results

Below is a summary of key themes which emerged from this process, together with key learnings and recommendations.

### Getting the balance right between information and counselling

Effective choice counselling requires a combination of information provision and counselling. We found an imbalance – with more emphasis on information-giving (e.g. the characteristics of respective methods) and less on opening communication to assist clients to assess their vulnerabilities, needs and options.

#### Key learning

More nuanced training on counselling and communication skills is required, with a focus on facilitating communication so that clients can reflect on their needs, circumstances, vulnerability and how this can inform their method choice.

### Healthcare providers’ own views can influence client’s choice

Although there was support for expanded options for PrEP, healthcare providers expressed misgivings about clients’ acceptance of a vaginal product. Concerns were expressed about efficacy, side effects and young clients’ ability to use PrEP as prescribed. There was acknowledgement that personally held views may influence communication about respective methods.

#### Key learning

In-service training needs to continuously reinforce how methods should be communicated to clients in a sensitive, evidence-guided, factual and balanced manner.

### Pre-exposure prophylaxis choice is new – are we on the right track?

Many healthcare providers expressed uncertainty about whether they were on the right track with providing PrEP choice counselling and some providers lacked confidence. The need for reassurance and guidance was expressed.

#### Key learning

This underscored the importance of ongoing support, reassurance and feedback beyond training when introducing new methods – where both confidence and competence are key.

### Factors influencing choice

Based on a prioritising activity, healthcare provider views on factors influencing client PrEP method choice varied among interviewees – highlighting the different factors that individuals value when selecting a PrEP method.

#### Key learning

This underscores the importance of client-centred counselling, understanding and addressing the personalised needs and preferences of clients in the decision-making process, reinforcing similar evidence from implementation research.^5,8^

### Job aids and demonstration models

The project used job aids developed by the Department of Health, which are provided as an addendum to the national DVR implementation guidelines.^14^ Healthcare providers mentioned that they found the job aids very helpful when explaining PrEP choices to clients; however, they requested that it is enlarged and summarised as a quick reference.

All sites have been provided with pelvic models to explain DVR use to clients, and although useful, it was felt that it may be intimidating to clients.

#### Key learning

It is important to revisit job aids and to make adaptations based on experience and feedback. More support on how best to use the pelvic model was indicated.

### The importance of personalised, face-to-face engagement

The importance of engagement with staff at the site level was underscored through this initiative. As described, the project is structured in such a way that the national office provides technical expertise and training (among other functions) to support project implementation. This process encouraged engagement with staff to reflect on their experiences with choice counselling in a participatory, supportive, non-threatening but honest manner. It is instructive to note that the importance of engaging with staff at site level is embodied in continuous quality improvement methodology where the value of leadership and management going to the actual location where people work is emphasised. This is known as ‘*Genchi Genbutsu*’ which translates as ‘go look, go see’.^15,16^

#### Key learning

The value of engagement with staff at site level for all aspects of project implementation has been reinforced. This is especially important with aspects of project implementation that are pivotal but less easy to define and measure such as the knowledge, attitude and skills involved in choice counselling and communicating about new biomedical HIV prevention methods and choice.

## Conclusion

In this report, we describe an iterative, participatory process which aimed to gain insight into healthcare providers’ implementation of choice counselling after training which identified gaps to guide the ongoing work of supervisors, project mentors and trainers.

The findings are supported by other studies looking at the importance of choice counselling as PrEP options expand and reinforce the point that the implementation thereof is a nuanced, complex process which needs to take into consideration respective PrEP methods’ specifications, benefits and drawbacks combined with the individual client’s needs and vulnerability.^6,8,17^

The use of the Transfer of Learning Matrix underscores the importance of following up training to gauge how knowledge and skills covered in training are interpreted and implemented on the ground.^9,10^ Healthcare providers need ongoing support to navigate ways to provide PrEP counselling which results in genuine informed decision-making. The process showed that once-off training and job aids for choice counselling are not enough and need to be supplemented by active engagement with healthcare providers on the ground to identify gaps and inform ongoing in-service training and mentoring. The value of engaging with staff at site level was also highlighted.

It is hoped that the methodology and insights gained through this process may be beneficial to others implementing PrEP and PrEP counselling, especially as new methods become available.

### Limitations

We acknowledge potential limitations, including social desirability bias due to interactive engagement with healthcare providers, since participants were drawn from project sites, and reliance on self-reported data. These factors may affect the accuracy of responses and limit the generalisability of findings beyond the study settings.
